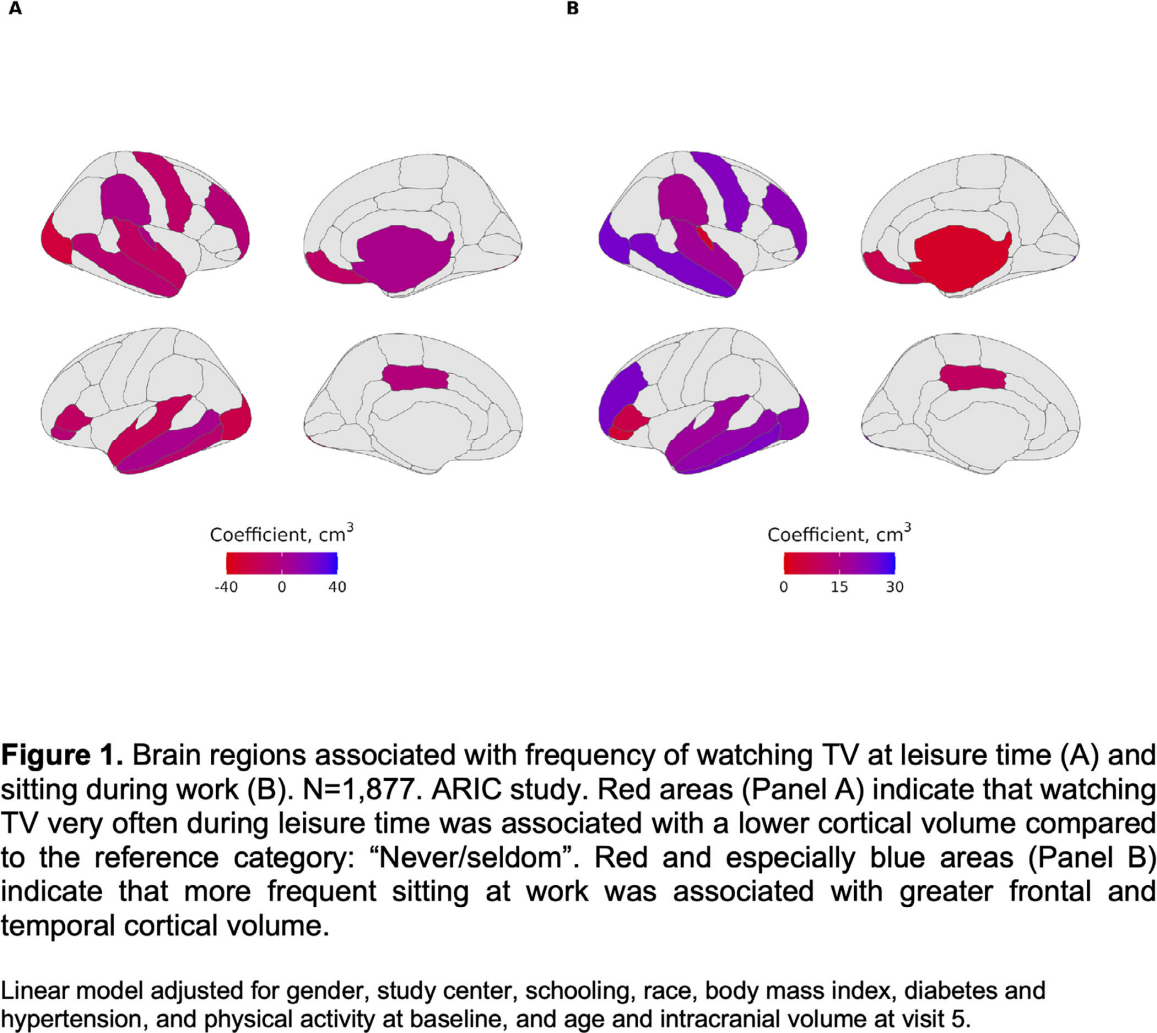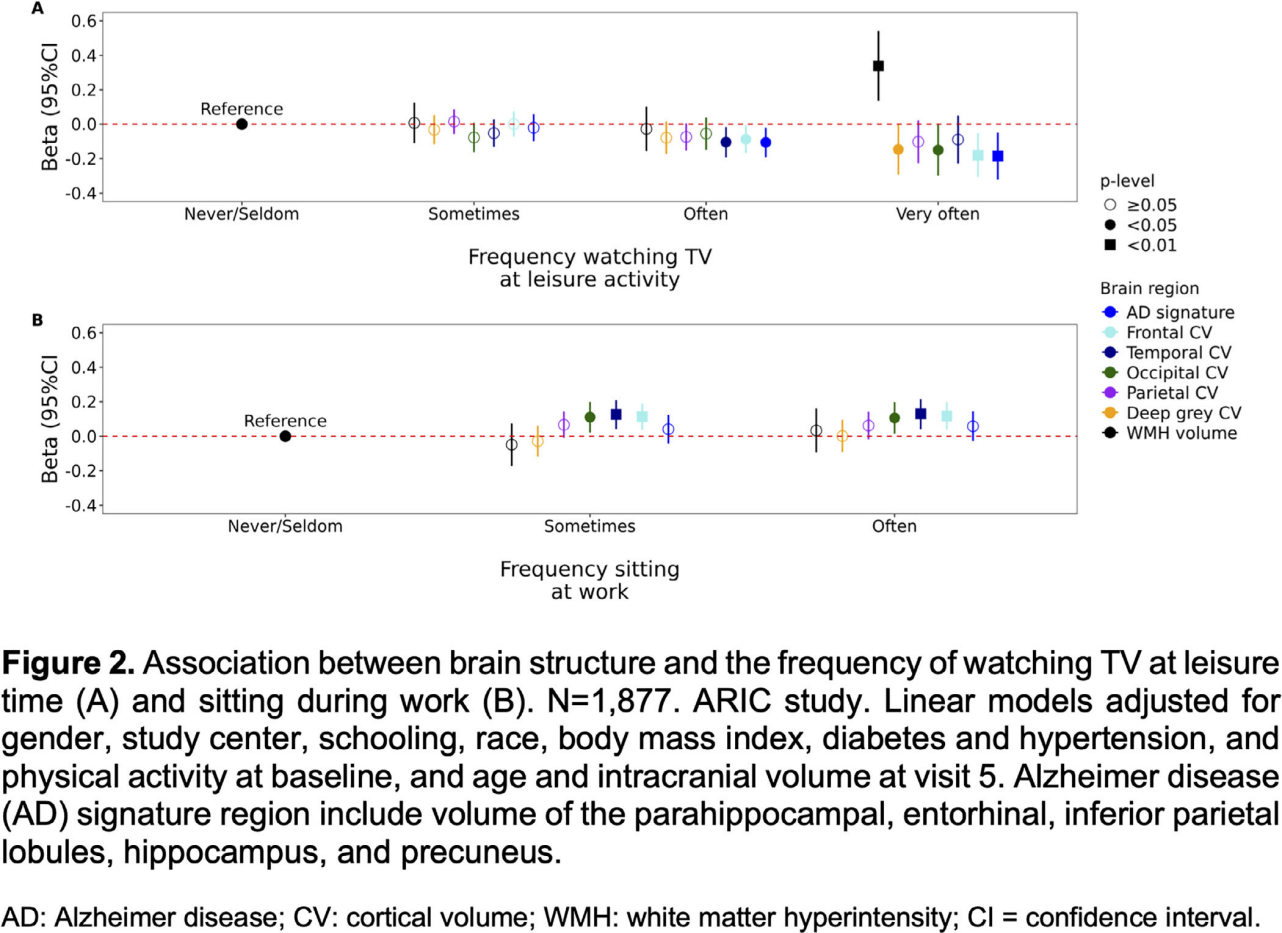# Associations between distinct sedentary behaviors with dementia risk and brain structure: findings of the ARIC Study

**DOI:** 10.1002/alz70860_103655

**Published:** 2025-12-23

**Authors:** Natan Feter, Anamika Nanda, Sarah Hourihan, Daniel Aslan, Jayne Feter, Bruce Duncan, Maria Inês Schmidt, M. Katherine Sayre, Pradyumna K. Bharadwaj, Madeline Ally, Hyun Song, Amit Amra, Silvio Maltagliati, Mark H.C. Lai, Rand R Wilcox, Yann C. Klimentidis, Gene Alexander, David A Raichlen

**Affiliations:** ^1^ University of Southern California, Los Angeles, CA, USA; ^2^ Universidade Federal do Rio Grande do Sul, Porto Alegre, Brazil; ^3^ Universidade Federal do Rio Grande do Sul, Porto Alegre, Rio Grande do Sul, Brazil; ^4^ Postgraduate Program in Epidemiology, Universidade Federal do Rio Grande do Sul, Porto Alegre, Rio Grande do Sul, Brazil; ^5^ Medical School, Universidade Federal do Rio Grande do Sul, Porto Alegre, Rio Grande do Sul, Brazil; ^6^ University of California Santa Barbara, Santa Barbara, CA, USA; ^7^ University of Arizona, Tuscon, AZ, USA; ^8^ University of Arizona, Tucson, AZ, USA

## Abstract

**Background:**

Longitudinal studies exploring the associations of sedentary behavior (SB) in different contexts with brain structure remain limited. We aimed to examine the association of SB at leisure and work with the risk of dementia and brain structure in middle‐aged and older adults over a 22‐year follow‐up.

**Method:**

We analyzed data from the Atherosclerosis Risk in Communities (ARIC) study. Participants were enrolled between 1987‐1989, with visit 5 between 2011‐2013. SB was measured as the frequency of watching television during leisure and sitting during work time over the 12 months prior to baseline (categorized as never/seldom, sometimes, often, or very often). We identified prevalent dementia cases based on NIA‐AA criteria using telephone interviews and medical record review. Brain structure was assessed during visit 5 using 3T MRI to quantify white matter hyperintensities (WMH) and regional cortical volumes estimated from Freesurfer. Poisson (dementia risk) and linear regression (brain structures) models were adjusted for sociodemographic, behavioral, and clinical variables in addition to total intracranial volume in structural analyses.

**Result:**

Participants (*N* = 5,747; 57% women) had a mean age of 52 (SD: 5.2) years at baseline. Participants who reported watching TV very often had a higher risk of dementia compared to those who watched TV never or seldom (RR: 1.69; 95%CI: 1.16, 2.45). In contrast, sitting often during work was linked to a lower risk (RR: 0.75; 95%CI: 0.57, 0.98). Watching TV very often was also associated with reduced cortical volume in the frontal (beta: ‐0.18; 95%CI: ‐0.30, ‐0.05), occipital (beta: ‐0.15; 95%CI: ‐0,30, ‐0.00), deep gray matter (beta: ‐0.15; 95% CI: ‐0.29, ‐0.00), and Alzheimer's disease signature regions (parahippocampal, entorhinal, inferior parietal lobules, hippocampus, and precuneus) (beta: ‐0.18; 95%CI: ‐0.31, ‐0.05) (Figure 1 and 2). Sitting during work often was linked to larger frontal (beta: 0.12; 95%CI: 0.04, 0.20) and temporal (beta: 0.13; 95%CI: 0.04, 0.22) cortical volumes. Participants who reported watching TV very often had increased WMH volumes (beta: 0.34; 95% CI: 0.13, 0.54) (Figure 2).

**Conclusion:**

The association of SB with the risk of dementia and brain structure is dependent of the cognitive demands involved in the behavior.